# Risk Factors of Reoperation in Patients with Intestinal Behçet’s Disease Treated by Initial Bowel Resection

**DOI:** 10.3390/jcm13226771

**Published:** 2024-11-11

**Authors:** Sun Jung Kim, Eun Ji Park, Hyeon Woo Bae, Yong Joon Lee, Min Young Park, Seung Yoon Yang, Yoon Dae Han, Min Soo Cho, Hyuk Hur, Joseph C. Carmichael, Byung Soh Min, Kang Young Lee

**Affiliations:** 1 Department of Surgery, Ajou University School of Medicine, Suwon 16499, Republic of Korea; mangoblow@gmail.com; 2 Graduate School of Medicine, Yonsei University College of Medicine, Seoul 03722, Republic of Korea; 3 Department of Surgery, Kyung Hee University School of Medicine, Seoul 02447, Republic of Korea; ejay.p2018@gmail.com; 4 Division of Colorectal Surgery, Department of Surgery, Yonsei University College of Medicine, Seoul 03722, Republic of Korea; incognito@yuhs.ac (H.W.B.); parkmy@yuhs.ac (M.Y.P.); syyy2000@yuhs.ac (S.Y.Y.); acylyoon@yuhs.ac (Y.D.H.); hhyuk@yuhs.ac (H.H.); bsmin@yuhs.ac (B.S.M.); kylee117@yuhs.ac (K.Y.L.); 5 Department of Surgery, CHA Bundang Medical Center, CHA University School of Medicine, Seongnam 13496, Republic of Korea; joonjoonlee@gmail.com; 6 Division of Colon and Rectal Surgery, Department of Surgery, School of Medicine, University of California, Irvine, CA 92697, USA; jcarmich@uci.edu

**Keywords:** intestinal Behçet’s disease, bowel resection, reoperation, risk factors, postoperative outcomes

## Abstract

**Background/Objectives**: Intestinal Behçet’s disease (iBD) often requires surgical intervention, with a significant proportion of patients needing reoperation. This study aimed to investigate the risk factors associated with reoperation in patients with iBD who underwent initial bowel resection and to evaluate the perioperative and long-term outcomes in these patients. **Methods**: This was a retrospective case-control study analyzing patients who underwent their initial bowel resection due to iBD between 2005–2021 at a tertiary referral hospital. Reoperation was considered a surgery due to postoperative complications (within 30 days of the initial surgery) or disease progression. **Results:** A total of 81 patients were included. The median follow-up duration was 107.1 months, during which 26 patients (32%) underwent reoperation. Multivariable analysis showed that the presence of hematological disorders (hazards ratio [HR], 9.13; 95% confidence interval [CI], 3.79–22.02, *p* < 0.001), higher c-reactive protein (CRP) levels before the initial surgery (HR, 1.01; 95% CI, 1.01–1.02, *p* < 0.001), and a shorter specimen resection length (HR, 0.96; 95% CI, 0.93–0.99, *p* = 0.011) were risk factors for reoperation. Patients who underwent reoperation had higher rates of postoperative complications (69.2% vs. 43.6%, *p* = 0.031), required longer antibiotic use (12 vs. 7 days, *p* = 0.012), and had extended hospital stays (18 vs. 9 days, *p* = 0.011). They also had worse 5-year survival rates than those who did not undergo reoperation (83.5% vs. 98.4%, *p* = 0.012). **Conclusions**: Concurrent hematological disorders, high preoperative CRP levels, and short specimen resection were associated with an increased risk of reoperation in patients with iBD who underwent their initial bowel resections. They also had worse perioperative and long-term outcomes.

## 1. Introduction

Behçet’s disease (Adamantiades–Behçet’s disease, BD) is a multisystemic inflammatory vascular disorder with chronic and recurrent occurrences [1]. It typically presents as oral aphthous ulcers, ocular inflammation, skin lesions, and genital ulcers, but it can also involve the joints and neurological, gastrointestinal, and cardiovascular systems [2]. Its etiology is indefinite but is related to genetics, infections, environmental factors, and immunological aberrations [3], affecting more males than females, with a general onset in the third decade of life [4].

Gastrointestinal manifestations of BD include symptoms presenting from the mouth to the anus, with an incidence of 2–60%, according to different regions [1]. In Northeast Asia, the incidence of BD is less than that of the Silk Road countries; however, the gastrointestinal manifestation of BD exceeds 7%, which is more than that of Turkey, Saudi Arabia, and Iraq [1,2]. Among the subtypes, intestinal Behçet’s disease (iBD) is one of the most common types, which frequently involves the ileocecal area apart from oral ulcers. Intestinal Behçet’s disease induces mucosal inflammation, ulcerations, ischemia, and infarction, and active interventions are required when it presents with bleeding, fistula, stricture, and perforation [2]. As with BD, treatment of iBD is initiated with 5-aminosalicylic acid, corticosteroids, immunomodulators, and anti-tumor necrosis factor alpha monoclonal antibody therapy [5]. However, recurrent or refractory iBD may require surgical intervention. More than 80% of patients with iBD are hospitalized, and one-fourth of them undergo surgery [6]. Even after surgical management, 30% and 47% of patients show clinical recurrence or disease progression between 2 and 5 years, respectively, and 12% and 22% need reoperations between 2 and 5 years, respectively [7]. In addition, although rare, there have been reports of gastrointestinal incomplete remission in some iBD patients who are refractory to medical managements [8].

The risk factors for recurrence after surgery have been reported diversly as follows: preoperative or intraoperative endoscopic findings (volcano-type ulcerations), high perioperative c-reactive protein (CRP) and erythrocyte sedimentation rate (ESR) levels, emergency surgeries, perioperative anti-BD medications, and intestinal perforations, etc. [9]. Meanwhile, the risk factors for reoperation in patients with iBD patients have been reported in several retrospective studies, but the results also have been inconsistent. Therefore, this study aimed to examine the risk factors of reoperation and explore clinical characteristics and perioperative outcomes of patients with iBD who underwent their initial surgeries.

## 2. Materials and Methods

### 2.1. Study Population

This was a retrospective case-control study that collected data until June 2024 from patients who underwent surgery for iBD in a tertiary, private teaching hospital (Severance Hospital, Seoul, Republic of Korea) between January 2005 and March 2023. Eligible patients were those aged >18 years who had undergone surgery with a diagnosis of BD. Patients whose diagnosis was not iBD, those who did not undergo initial bowel surgery, and those who did not undergo bowel resection were excluded. Patient consent was waived due to the retrospective nature of the study. Ethical approval for this study was provided by the Institutional Review Board of the Severance Hospital, Yonsei university, Seoul, Republic of Korea, on 16 July 2023 (IRB No. 4-2023-0658). The study was performed in compliance with the STROBE and STROCSS guidelines [10,11].

### 2.2. Definition of Variables

Preoperative blood test results, such as serum CRP, albumin, and hemoglobin (Hb), were collected, and the date of the test was nearest to the date of the surgery and within one week from it at the same time. Preoperative anti-BD medications were subdivided into multiple, single, and none. Previous hematological disorders included preoperative diagnosis of myelodysplastic syndrome (MDS) or aplastic anemia. Indications for initial bowel resection included medical intractability, intraabdominal abscess or fistula, bowel stenosis, gastrointestinal bleeding, bowel perforation, and others.

Postoperative complications were collected for those occurring within 30 days of surgery. The anastomosis configuration was subgrouped into end-to-end, end-to-side, and side-to-side, while the anastomosis type was subgrouped into staple-assisted and hand-sewn groups. The staple-assisted group was categorized as such if at least one stapler was used for the anastomosis. The anastomosis type for patients who underwent stoma repair without anastomosis was determined from that of subsequent stoma repair surgery.

### 2.3. Outcomes

Reoperations included those performed for postoperative complications (30 days within surgery) and for clinical relapse or disease progression despite postoperative anti-BD medication. Simple stoma repair was not regarded as a reoperation.

Perioperative outcomes such as operative characteristics, pathologic characteristics, or postoperative characteristics were collected, as well as long-term outcomes.

### 2.4. Statistical Analysis

Statistical analyses were performed using SPSS 26.0, and statistical significance was set at *p*-value < 0.05. Clinicopathological and perioperative characteristics were analyzed using the chi-square test or Fisher’s exact test for categorical variables and Student’s *t*-test for quantitative variables. Risk analysis for reoperation was performed using Cox regression analysis with forward variable selection. The cutoff measure of quantitative variables for risk analysis, such as the mean or median, was determined as a representative value. The overall 5-year reoperation-free survival and survival analyses were analyzed using a Kaplan–Meier estimation and a log-rank test. All categorical data were complete, and the quantitative variables were analyzed, excluding missing data.

## 3. Results

### 3.1. Patient Characteristics

**Figure 1:**
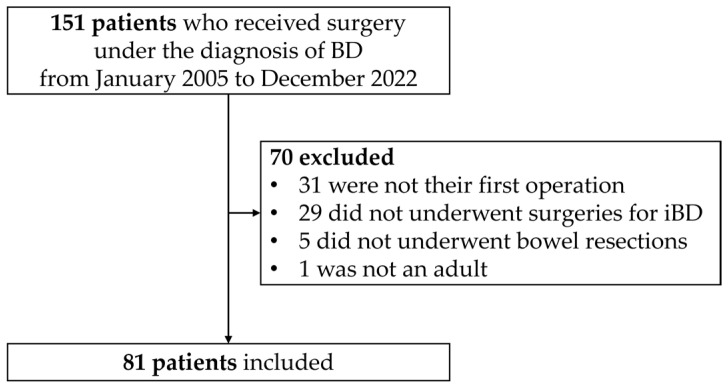
Flowchart of patient selection. BD: Behçet’s disease; iBD: intestinal Behçet’s disease.

A total of 151 patients were pooled and 81 patients were included after excluding 70 patients (Figure 1). All patients were Asian. The mean ages at diagnosis of BD, diagnosis of iBD, and initial bowel resection were 39.4, 43.7, and 46.9 years, respectively (Table 1). Most patients were female (65.4%), and 21% had concurrent hematological disorders. Medical intractability was the most common reason for the initial bowel resection, accounting for 65.4% of the cases (Table 1).

When comparing the patients who underwent reoperation to those who did not after their initial bowel resections, it was observed that the reoperation group had lower BMIs before the initial surgery (19.6 ± 4.2 vs. 21.5 ± 3.6 kg/m^2^, *p* = 0.039), a higher prevalence of hematological disorders (46.2% vs. 9.1%, *p* < 0.001), and elevated median CRP levels prior to the initial surgery (84.7 vs. 46.3 mg/dL, *p* = 0.004) (Table 1).

### 3.2. Perioperative Outcomes

An emergency operation was conducted in 19 patients (23.5%) (Table 2). Patients who underwent reoperation had undergone segmental resection of the small bowel more often during their initial surgery, whereas the patients who did not undergo reoperation had undergone ileocecectomy, right hemicolectomy, or total colectomy more often (*p* = 0.041). The patients who underwent reoperation required longer median antibiotic use (12 vs. 7 days, *p* = 0.012) and had extended median hospital stays (18 vs. 9 days, *p* = 0.011). They also exhibited higher rates of overall postoperative complications (69.2% vs. 43.6%, *p* = 0.031), specifically, anastomosis leakage (7.7% vs. 0%, *p* = 0.037) and wound infections (38.5% vs. 5.5%, *p* < 0.001). A stoma was created in only three patients without anastomosis. There were no significant differences in the pathologic characteristics between the two groups (Table 2).

### 3.3. Reoperation

**Figure 2:**
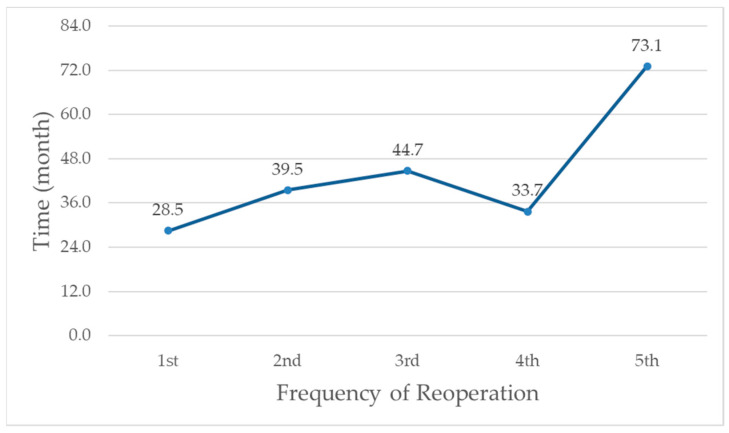
Mean time from initial surgery to reoperation by frequency of reoperations in 26 patients.

The median follow-up duration was 107.1 months, during which 26 patients (32%) underwent reoperation, including 3 patients (3.7%) due to postoperative complications and 23 patients (28.4%) due to disease progression. Among them, 9 (11.1%), 18 (22.2%), and 22 (27.2%) patients underwent reoperation within 1, 3, and 5 years after their initial operations. The reason for the first reoperation included medical intractability (53.8%), acute perforation (15.4%), subacute perforation (19.2%), hemorrhage (3.8%), and stricture (7.7%). Previous anastomosis involvement was found in 18 patients (69.2%). Two patients underwent a total of five additional reoperations during surveillance. The mean times from initial surgery to the first, second, third, fourth, and fifth reoperation were 28.5, 39.5, 44.7, 33.7, and 73.1 months, respectively (Figure 2). The reason the average time to the fourth reoperation was shorter than to the third is that half of the four patients underwent a fourth reoperation within one year from the initial surgery.

Univariate risk analysis using Cox regression showed that low BMI, the presence of hematological disorders, high CRP levels before the initial surgery, low Hb and albumin levels before the initial surgery, prolonged postoperative antibiotics use and hospital stay, postoperative complications, and pathologic necrosis of the specimen were risk factors for reoperation (Table 3). However, the multivariable analysis showed that the presence of hematological disorders (hazards ratio [HR], 9.13; 95% confidence interval [CI], 3.79–22.02, *p* < 0.001), higher CRP levels before the initial surgery (HR, 1.01; 95% CI, 1.01–1.02, *p* < 0.001), and a shorter specimen resection length were risk factors for reoperation (HR, 0.96; 95% CI, 0.93–0.99, *p* = 0.011).

**Figure 3:**
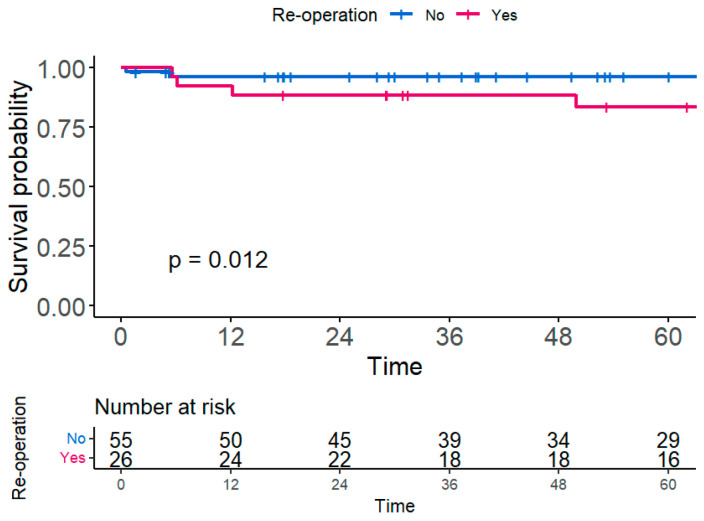
Five-year overall survival according to patients who underwent reoperation or not.

Of all patients, eight (9.9%) deaths occurred, five of whom died from postoperative infection, and the rest died from short bowel syndrome, septic colitis, and gastric BD bleeding. Patients who underwent reoperation had worse 5-year survivals than those who did not undergo reoperation (reoperation vs. no reoperation: 83.5% vs. 98.4%, *p* = 0.012) (Figure 3).

## 4. Discussion

In the current study, an accompanying hematological disorder, elevated CRP levels before initial surgery, and a longer specimen resection length were significantly associated with reoperation in patients with iBD. Four previous retrospective studies reported risk factors for reoperation in patients with iBD, all of which were performed at the same hospital as the current study (Table 4) [7,12,13,14]. The most frequently mentioned risk factor for reoperation in the previous studies was postoperative steroid use. However, our study did not include it in the analysis because it was unclear from when the addition of medication in relation to the surgery would be considered as postoperative anti-BD medication. Preoperative serum ESR, which was identified as a risk factor in Park’s study, was also excluded from our analysis because most patients had not been tested for it. Additionally, endoscopic findings were available for 71 out of 81 patients, but because the average duration between the examination and the first surgery was 8 months, with a maximum of 39 months, they were not included in the analysis. Other variables, such as a lower BMI and postoperative complications, previously reported as risk factors for reoperation in iBD patients, were associated with reoperation in the current study’s univariate analysis but not in the multivariate analysis, probably due to the effect of hematological disorders.

In the current study, the presence of hematological disorders had the greatest impact on reoperation (HR, 9.13; 95% CI, 3.79–22.02). Out of the 17 patients who had concurrent hematological disorders, 13 (76.5%) had MDS. BD is often accompanied by hematological disorders, including MDS, and it usually presents with trisomy 8 [15]. In a systematic review performed by Handa et al. [16], 41 out of 60 patients (66%) with BD complicated by MDS had intestinal lesions. Kanamitsu et al. had reported five cases of pediatric iBD complicated by myeloid malignancies, suggesting the role of TNF-α in both diseases [17]. A recent multicenter retrospective study reported that, as compared to 70 matched patients with iBD alone, 35 iBD patients complicated with MDS required more surgical treatment (51.4% vs. 24.3%, *p* = 0.010), showed poorer responses to both medical (including anti-TNF- α agents) and surgical treatments (75.0% vs. 11.4%, *p* < 0.001), and had a higher mortality rate (28.6% vs. 0%, *p* < 0.001) [18]. They also reported that seven iBD-MDS patients underwent hematopoietic stem cell transplantation (HSCT), and all of them, including four who had not responded to medical treatement prior to HSCT, achieved complete remission of iBD. In the current study, reoperation was performed a maximum of five times, with 46.2%, 61.5%, 66.7%, 75.0%, and 50.0% of patients undergoing one, two, three, four, and five reoperations, respectively, due to hematological disorders. Considering this, HSCT might be beneficial for iBD patients with concurrent hematological disorders who are refractory to surgical treatments.

Higher preoperative CRP levels have also been identified as a risk factor for reoperation after initial bowel surgery in patients with IBD in the current study. In this study, we found that each 10 mg/L increase in preoperative CRP levels raised the odds of reoperation by 10.1 times. The study by Jung et al. also found that patients with preoperative CRP levels above 4.4 mg/dL (or 44 mg/L) had 46.85 times higher odds of reoperation as compared to those below this level [7]. In fact, preoperative CRP is a well-known risk factor associated with worse postoperative outcomes not only in iBD but in colorectal surgery in general [19]. However, in the univariate analysis of the current study, reoperation was not associated with the type of indication for the initial iBD surgery or with emergency operations. This leads us to speculate that preoperative CRP might reflect the disease activity in iBD, thus affecting the postoperative course of disease activity of the bowel. In fact, only 3 of the 26 reoperative patients required reoperation due to postoperative complications. Considering this, we assume that if preoperative baseline CRP had been included in the study conducted by Kang et al., who reported a strong correlation between higher postoperative CRP and worse postoperative outcomes, the association of preoperative CRP might have been highlighted over that of postoperative CRP. It is also noteworthy that in inflammatory bowel diseases such as Crohn’s disease, which often involves ileocolic lesions similar to iBD, preoperative CRP levels are associated with worse postoperative outcomes and complications [20].

The current study found that surgical techniques, whether the anastomosis was conducted with the hand-sewn method or a stapler, as well as the anastomosis configuration, did not influence the likelihood of reoperation. However, the resection length of the bowel was significantly associated with a reduced risk of reoperation (HR, 0.96; 95% CI, 0.93–0.99), meaning that for every additional 10 cm of bowel resection, the risk of reoperation was reduced by 10%. In Crohn’s disease, transmural inflammation and intestinal stenosis or fistulas are common pathologies. Consequently, surgical techniques such as the Kono-S anastomosis, which widen the lumen, are often reported to be useful in preventing reoperation [21,22]. However, considering that only 7.7% of the pathologies observed after reoperation were stricturing in this study, it can be inferred that, rather than the anastomosis technique itself, extending the resection to remove visibly affected areas might be a more effective approach for preventing reoperation.

A limitation of this study is that it was a retrospective study performed in a single institution. Nevertheless, this study is significant because of the rarity of this disease and is unique in that it analyzed only patients who underwent their initial bowel resection surgery, using the most recent data.

## 5. Conclusions

Concurrent hematological disorders, high preoperative CRP levels, and short specimen resection were associated with an increased risk of reoperation in patients with iBD who underwent their initial bowel resection. They also had worse perioperative and long-term outcomes.

## Figures and Tables

**Table 1:** Preoperative characteristics of patients who underwent their initial bowel resections due to iBD.

	Overall (*n* = 81)	Reoperation Group (*n* = 26)	Non-Reoperation Group (*n* = 55)	*p*-Value
Sex				0.621
Male	28 (34.6%)	8 (30.8%)	20 (36.4%)	
Female	53 (65.4%)	18 (69.2%)	35 (63.6%)	
Age at diagnosis of BD (years)	39.4 ± 13.0	38.8 ± 12.5	39.7 ± 13.4	0.755
Age at diagnosis of iBD (years)	43.7 ± 12.2	43.4 ± 13.4	43.8 ± 11.7	0.877
Age at initial surgery (years)	46.9 ± 12.3	45.6 ± 13.2	47.5 ± 12.0	0.537
Time to initial surgery from diagnosis (month)	16.8 (3.4, 51.7)	15.1 (3.3, 3.8)	16.8 (3.3, 64.7)	0.479
BMI at initial surgery (kg/m^2^)	20.9 ± 3.8	19.6 ± 4.2	21.5 ± 3.6	0.039
Hematological disorder	17 (21.0%)	12 (46.2%)	5 (9.1%)	<0.001
Operative history	14 (17.3%)	5 (19.2%)	16 (29.1%)	0.344
Drugs used before initial surgery				0.627
Multiple	56 (69.1%)	17 (65.4%)	39 (70.9%)	
Single	19 (23.5%)	6 (23.1%)	13 (23.6%)	
None	6 (7.4%)	3 (11.5%)	3 (5.5%)	
Indication for initial surgery				0.420
Medical intractability	53 (65.4%)	18 (69.2%)	35 (63.6%)	
Abscess/fistula	3 (3.7%)	1 (3.8%)	2 (3.6%)	
Stenosis	4 (4.9%)	1 (3.8%)	3 (5.5%)	
Bleeding	6 (7.4%)	0 (0%)	6 (10.9%)	
Perforation	13 (16.0%)	6 (23.1%)	7 (12.7%)	
Other	2 (2.5%)	0 (0%)	2 (3.6%)	
ASA score				0.499
1–2	57 (70.4%)	17 (65.4%)	40 (72.7%)	
3–4	24 (29.6%)	9 (34.6%)	15 (27.3%)	
CRP before initial surgery(mg/dL)	54.4 (20, 109)	84.7 (39.1, 168.8)	46.3 (14.6, 80.5)	0.004
Albumin before initial surgery (g/dL)	3.4 ± 0.6	3.2 ± 0.6	3.5 ± 0.7	0.066
Hb before initial surgery (g/dL)	10.1 ± 1.5	9.6 ± 1.4	10.3 ± 1.6	0.052

iBD: intestinal Behçet’s disease; BMI: body mass index; ASA: American Society of Anesthesiologists; CRP: C-reactive protein; Hb: hemoglobin.

**Table 2:** Perioperative characteristics of the patients who underwent their initial bowel resection due to iBD.

	Overall (*n* = 81)	Reoperation Group (*n* = 26)	Non-Reoperation Group (*n* = 55)	*p*-Value
Emergency operation	19 (23.5%)	8 (30.8%)	11 (20.0%)	0.286
Open conversion	2 (2.5%)	0 (0%)	2 (3.6%)	>0.999
Operation name				0.041
Ileocecectomy	51 (63.0%)	15 (57.7%)	36 (65.5%)	
Right hemicolectomy	22 (27.2%)	6 (23.1%)	16 (29.1%)	
Segmental resection of small bowel	6 (7.4%)	5 (19.2%)	1 (1.8%)	
Total colectomy	2 (2.5%)	0 (0%)	2 (3.6%)	
Operative method				0.383
Open	20 (24.7%)	8 (30.8%)	12 (21.8%)	
Laparoscopic	61 (75.3%)	18 (69.2%)	43 (78.2%)	
Operation time (min)	142 (111, 192)	152 (122, 193)	135 (107, 192)	0.227
Intraoperative blood loss (mL)	20 (0.100)	35 (0, 125)	10 (0, 100)	0.319
Anastomosis type				0.767
End-to-end	12 (14.8%)	5 (19.2%)	7 (12.7%)	
End-to-side	55 (67.9%)	17 (65.4%)	38 (69.1%)	
Side-to-side	14 (17.3%)	4 (15.4%)	10 (18.2%)	
Anastomosis method				0.953
Hand-sewn	37 (45.7%)	12 (46.2%)	25 (45.5%)	
Stapler-assisted	44 (54.3%)	14 (53.8%)	30 (54.5%)	
Multiple anastomosis	2 (2.5%)	1 (3.8%)	1 (1.8%)	0.542
Stoma formation	3 (3.7%)	2 (7.7%)	1 (1.8%)	0.240
Postoperative antibiotics use (days)	8 (5, 16)	12 (7, 26)	7 (5, 13)	0.012
Postoperative hospital stay (days)	10 (7, 22)	18 (8, 33)	9 (7, 16)	0.011
Postoperative complication				
Overall	42 (51.9%)	18 (69.2%)	24 (43.6%)	0.031
Intra-abdominal infection	14 (17.3%)	7 (26.9%)	7 (12.7%)	0.115
Anastomosis leak	2 (2.5%)	2 (7.7%)	0 (0%)	0.037
Bleeding	3 (3.7%)	0 (0%)	3 (5.5%)	0.547
Ileus	7 (8.6%)	3 (11.5%)	4 (7.3%)	0.675
Colitis	11 (13.6%)	6 (23.1%)	5 (9.1%)	0.086
Wound infection	13 (16.0%)	10 (38.5%)	3 (5.5%)	<0.001
Other	15 (18.5%)	7 (26.9%)	10 (18.2%)	0.367
Grade				0.036
I	7 (8.6%)	3 (11.5%)	4 (7.3%)	
II	20 (24.7%)	10 (38.5%)	10 (18.2%)	
IIIa	4 (4.9%)	1 (3.8%)	3 (5.5%)	
IIIb	5 (6.2%)	3 (11.5%)	2 (3.6%)	
IV	1 (1.2%)	1 (3.8%)	0 (0%)	
V	1 (1.2%)	0 (0%)	1 (1.8%)	
Pathology of specimen				0.503
Inflammation	3 (3.7%)	1 (3.8%)	0 (3.6%)	
Ulcer	49 (60.5%)	14 (53.8%)	35 (63.6%)	
Necrosis	1 (1.2%)	1 (3.8%)	0 (0%)	
Perforation	28 (34.6%)	10 (38.5%)	18 (32.7%)	
Resection length (cm)	29.5 ± 17.2	26.0 ± 13.8	31.2 ± 18.4	0.208
Maximum luminal diameter (cm)	7.4 ± 2.4	7.5 ± 2.4	7.3 ± 2.4	0.852

iBD: intestinal Behçet’s disease.

**Table 3:** Risk factors for reoperation after the initial bowel resection in patients with iBD.

	Univariable Analysis		Multivariable Analysis	
	HR (95% CI)	*p*-Value	HR (95% CI)	*p*-Value
Sex (Male)	1.37 (0.59–3.16)	0.459		
Age at initial surgery (years)	1 (0.97–1.03)	0.976		
BMI at initial surgery (kg/m^2^)	0.87 (0.78–0.97)	0.016		0.071
ASA score (3–4)	1.83 (0.81–4.14)	0.144		
Hematological disorder	6.21 (2.83–13.63)	<0.001	9.13 (3.79–22.02)	<0.001
Operative history	0.64 (0.24–1.71)	0.376		
Drugs used before initial surgery (None)		0.262		
Single	0.37 (0.09–1.52)	0.168		
Multiple	0.36 (0.1–1.24)	0.105		
Indication for initial surgery (Medical intractability)		0.987		
Abscess/fistula	0.89 (0.12–6.67)	0.909		
Stenosis	0.54 (0.07–4.02)	0.543		
Bleeding	0 (0–0)	0.978		
Perforation	1.22 (0.48–3.07)	0.680		
Other	0 (0–0)	0.989		
CRP before initial surgery (mg/L)	1.01 (1–1.01)	<0.001	1.01 (1.01–1.02)	<0.001
Albumin before initial surgery (g/dL)	0.48 (0.26–0.91)	0.023		0.077
Hb before initial surgery (g/dL)	0.73 (0.56–0.94)	0.016		0.557
Indication for initial surgery (Medical intractability)		0.289		0.441
Perforations	1.16 (0.48–2.78)	0.136	0.29 (0.03–2.9)	0.293
Others	0.22 (0.03–1.62)	0.745	0.21 (0.01–5.08)	0.337
Emergency operation	1.37 (0.59–3.17)	0.461		
Open conversion	0.05 (0–954.6)	0.547		
Operation name (Ileocecectomy)		0.153		
Right hemicolectomy	0.94 (0.36–2.43)	0.901		
Segmental resection of small bowel	3.08 (1.12–8.49)	0.030		
Total colectomy	0 (0–0)	0.986		
Operative method (Open)	1.06 (0.46–2.46)	0.884		
Operation time (min)	1.00 (0.99–1.01)	0.904		
Intraoperative blood loss (mL)	1.00 (1.00–1.00)	0.523		
Anastomosis type (End-to-end)		0.893		
End-to-side	0.85 (0.31–2.31)	0.744		
Side-to-side	0.73 (0.19–2.72)	0.636		
Anastomosis method (Stapler-assisted)	1.03 (0.48–2.23)	0.944		
Multiple anastomosis	1.16 (0.16–8.61)	0.883		
Stoma formation	3.26 (0.74–14.28)	0.117		
Postoperative antibiotics use (days)	1.02 (1.01–1.03)	<0.001		0.090
Postoperative hospital stay (days)	1.02 (1.01–1.03)	<0.001		0.106
Postoperative complication	2.46 (1.07–5.67)	0.034		0.229
Pathology of specimen (inflammation)		0.018		0.848
Ulcer	0.46 (0.06–3.61)	0.460		0.665
Perforation	0.50 (0.06–4.08)	0.521		0.847
Necrosis	39.1 (1.38–1113.10)	0.032		0.391
Resection length (cm)	0.99 (0.96–1.01)	0.310	0.96 (0.93–0.99)	0.011
Maximum luminal diameter (≥5 cm)	0.99 (0.85–1.16)	0.904		

iBD: intestinal Behçet’s disease; BMI: body mass index; ASA: American Society of Anesthesiologists; CRP: C-reactive protein; Hb: hemoglobin.

**Table 4:** Previous studies reporting risk factors of reoperation in patients with intestinal Behçet’s disease.

First Author/s, Year	Inclusion Period (Year)	Inclusion Criteria	Patients (N)	Outcomes	Risk Factors
Jung et al., 2011 [7]	1986–2010	All surgical patients	72	Reoperation	Volcano-shaped ulcer, higher preoperative CRP, postoperative steroids use
Baek et al., 2015 [12]	1995–2012	All surgical patients	91	Reoperation	Postoperative steroid use, postoperative complications, lower BMI
Park et al., 2017 [13]	2006–2016	Patients with repeated surgery	41	Early reoperation (<6 months)	Initial emergency operation, higher ESR
Kang et al., 2021 [14]	2005–2018	All surgical patients	90	Reoperation	Higher postoperative CRP, perioperative steroids use

CRP: C-reactive protein; ESR: erythrocyte sedimentation rate.

## Data Availability

The data supporting the findings of this study are available from the corresponding author upon reasonable request.
